# Haplotype Analyses of Haemoglobin C and Haemoglobin S and the Dynamics of the Evolutionary Response to Malaria in Kassena-Nankana District of Ghana

**DOI:** 10.1371/journal.pone.0034565

**Published:** 2012-04-10

**Authors:** Anita Ghansah, Kirk A. Rockett, Taane G. Clark, Michael D. Wilson, Kwadwo A. Koram, Abraham R. Oduro, Lucas Amenga-Etego, Thomas Anyorigiya, Abraham Hodgson, Paul Milligan, William O. Rogers, Dominic P. Kwiatkowski

**Affiliations:** 1 Noguchi Memorial Institute for Medical Research, University of Ghana, Accra, Ghana; 2 Wellcome Trust Centre for Human Genetics, Oxford, United Kingdom; 3 London School of Hygiene and Tropical Medicine, London, United Kingdom; 4 Navrongo Health Research Centre, Navrongo, Ghana; 5 Naval Medical Research Unit #2, Jakarta, Indonesia; 6 Wellcome Trust Sanger Centre, Hinxton, United Kingdom; London School of Hygiene and Tropical Medicine, United Kingdom

## Abstract

**Background:**

Haemoglobin S (HbS) and C (HbC) are variants of the *HBB* gene which both protect against malaria. It is not clear, however, how these two alleles have evolved in the West African countries where they co-exist at high frequencies. Here we use haplotypic signatures of selection to investigate the evolutionary history of the malaria-protective alleles HbS and HbC in the Kassena-Nankana District (KND) of Ghana.

**Methodology/Principal Findings:**

The haplotypic structure of HbS and HbC alleles was investigated, by genotyping 56 SNPs around the *HBB* locus. We found that, in the KND population, both alleles reside on extended haplotypes (approximately 1.5 Mb for HbS and 650 Kb for HbC) that are significantly less diverse than those of the ancestral HbA allele. The extended haplotypes span a recombination hotspot that is known to exist in this region of the genome

**Significance:**

Our findings show strong support for recent positive selection of both the HbS and HbC alleles and provide insights into how these two alleles have both evolved in the population of northern Ghana.

## Introduction

Haemoglobin S or “sickle Haemoglobin" (HbS) – rs334 and Haemoglobin C (HbC) – rs33930165 are glutamic acid→valine and glutamic acid→lysine substitutions, respectively, at codon six of the *HBB* gene encoding the β-globin component of haemoglobin. HbS and HbC result from substitutions at the second and first position of *HBB* codon six, respectively. HbS is distributed widely throughout sub-Saharan Africa, as well as parts of the Middle East and is maintained at about 10% frequency in many malaria endemic regions [1], [2]. HbC is less widely distributed, found mainly in the northern savannas of West Africa around Mali, Burkina Faso and Ghana [3], [4], [5]. The HbC variant results in a less severe clinical phenotype than the sickle-cell disease caused by the HbS homozygous state. Individuals homozygous for HbC have a relatively mild haemolytic anaemia, and heterozygotes rarely experience significant anaemia [6].

Over half a century ago, Haldane [7] and Allison [8] proposed that the high frequencies of haemoglobinopathies such as thalassaemia and sickle cell disease were the result of balancing selection in response to malaria (“The malarial hypothesis"). Abundant evidence exists to support HbS as a classic example of balanced polymorphism in human populations, offering 10-fold reduced risk of severe malaria in the heterozygous state [9], [10], and thus persisting in the population despite the deleterious effect of the homozygous state. The evidence for a beneficial effect of HbC is more recent, and is based on the observation that HbC heterozygotes were protected against severe malaria in the Dogon ethnic group of Mali [3], and in the Mossi ethnic group of Burkina Faso, where HbC was associated with a 29% reduction in risk of clinical malaria in the heterozygote and 93% in the homozygous states [4]. Thus HbC confers weaker protection than HbS in the heterozygous state but, in contrast to HbS, homozygotes appear to enjoy strong protection against malaria without substantial loss of fitness [4], [11], [12].

We still have a poor understanding of the evolutionary factors that have led to the distinctive geographical and ethnic distributions of the HbC and HbS alleles. The KND region of Northern Ghana provides an interesting example of a population where both alleles co-exist, thus providing an opportunity to compare their evolution within a single population. Here we test for recent positive selection of the HbS and HbC alleles within this population by genotyping 56 SNPs around this locus and looking for evidence of extended haplotypes, noting that this region of the genome contains a well-known recombination hot spot.

## Materials and Methods

### Ethical approvals

This study was based on a case-control study of severe malaria at the Navrongo War Memorial Hospital and four health centres in the Kassena-Nankana district of Ghana, as described elsewhere [13] [14]. It was approved by the scientific and ethical review boards of the Noguchi Memorial Institute for Medical Research, the Navrongo Health Research Center, and US Naval Medical Research Unit #3, with written informed consent for genetic analyses on these the samples.

### Sample Selection

We performed HbS and HbC genotyping on 806 population control samples, and found HbS and HbC allele frequencies were 0.038 and 0.128 respectively (Table 1). The population controls consisted of 2 major ethnic groups (Kassem 63.0%, Nankan 33.4%, other 3.6%), in which we observed no differences in allele frequency for both HbS and HbC (Table 1). Based on these genotyping data we identified an informative set of 201 individuals for analysis in this study, comprising 20 instances of the HbS allele, 53 instances of the HbC allele, and 329 instances of the HbA allele (Table 2).

**Table 1:** Allele frequencies of HbS and HbC in the different ethnic groups observed in the control group.

Ethnic group	n	HbS MAF*	HbC MAF**
kassem	508	0.030	0.106
Nankan	269	0.032	0.104
Buli	15	0.000	0.250
Other	14	0.154	0.136
Overall	806	0.038	0.128

* genotyping success 97.8%, HWE-P 0.114;

** genotyping success 97.8%, HWE-P 0.738; MAF-minor allele frequency, HWE-P - probability from a chi-square Hardy-Weinberg Equilibrium.

**Table 2:** Description and distribution of the chromosomes in the selected samples used in the analysis.

	genotypes					
	AA	AG	GG/AA	AT	TT	total
Description	HbC homozygous	HbC heterozygous	HbA homozygous	HbS heterozygous	HbS homozygous	
Individuals (201)	15	23	145	16	2	
Chromosomes						
HbA	-	23	290	16	-	329
HbC	30	23	-	-	-	53
HbS	-	-	-	16	4	20
total						402

Table 2 is a description of the genotypes of the three haemoglobin variants analysed and the number of chromosomes analysed for each variant.

### Marker selection

**Figure 1:**
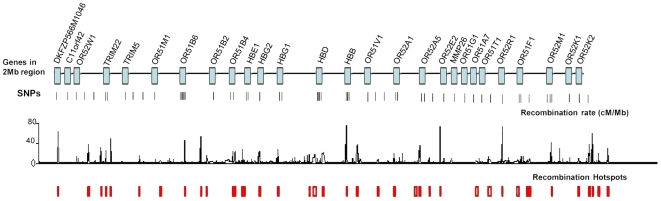
A schematic diagram of the 2 Mb region of chromosome 11 studied. Figure 1 indicates the genes spanning the 2 Mb of chromosome 11 studied and the spacing of the 56 SNP genotyped. Recombination rates between markers within the region and locations of recombination ‘hot spots’ (www.Hapmap.org) are included.

We genotyped the chosen samples for 56 markers spanning a 2 Mb region on both the 5′ (25 markers) and 3′ (30 markers) ends of the HbC/HbS loci in the *HBB* gene cluster found on 11p15.5. These markers had been identified using the HapMap project database (Yoruba population YRI, release 22), complemented by in-house capillary sequencing of a 110 Kb region around HbS/HbC in the Gambian population [15] and from available sequences on a study of the effects of recombination hotspots on selection in HbC alleles [16]. A description of marker selection is shown in Figure 1 and a full list of the 56 markers with details for their reference cluster ID numbers (rs#), chromosomal positions and locations of recombination hotspots are shown in Table S1. Available sequences from a study of the effects of recombination hotspots on the selection of HbC allele, [15], were aligned with Gene Ensembl release (30) sequences to design assays for 5 of their SNPs identifying core HbC haplotypes. Although the HapMap recombination hotspot data places HbS and HbC within the 3 kb hotspot (chromosomal position 5204001-5207001), the hot spot identified by Chakravarti and colleagues [17], by sequencing a 5.2 kb region spanning the *β*-globin gene places the HbC/HbS mutations about 500 bps away from the hotspot [16].

### Genotyping

Briefly, genomic DNA samples [13] were subjected to whole-genome amplification (WGA) using the primer extension pre-amplification (PEP) [18]. Genotyping was performed using SEQUENOM® hME Mass-Array® technology (www.sequenom.com). Assays were designed using the SpectroDESIGNER® (Sequenom) software. Two microlitres of a 20-fold dilution of PEP product was used per 5 ul typing reaction [19]. The 56 assays were grouped into 14 multiplexes. SNPs with greater than 20% missing data were excluded, resulting in a final set of 51 SNPs for analysis. SNP identifiers, coordinates and genotyping success rates are documented in Table S2. All genotyping data is available for download (Table S3).

### Simulation of a KND Haplotype Population

Since the 201 individuals that we genotyped were enriched for the HbC and HbS alleles, we sought to overcome sampling bias by simulating a population of 1000 haplotypes based on the known allele frequencies of HbC (12.8%), HbS (3.8%) and HbA (83.4%) in the general population i.e. “the community control group" (Table 1). In particular, we used a permutation approach to simulate haplotypes comprising 51 SNPs used for the final analysis.

### Statistical analysis

Deviations from Hardy-Weinberg equilibrium were assessed using a Chi-square one degree of freedom statistical test. Haplotypes were inferred using fastPHASE (version 1.2) software package [20] and sorted by the loci of the two *HBB* gene variants. We used the extended haplotype homozygosity (EHH) measure to compare the ancestral allele HbA with the derived HbS and HbC alleles. HbS and HbC were set as the core haplotype SNPs for measuring the EHH scores within the 2 Mb region and represented as bifurcation diagrams and EHH plots. EHH was measured using web-based software, SWEEP (http://www.broadinstitute.org/mpg/sweep/).

The distribution-free property of permutation re-sampling method was explored to measure differences/heterozygosity and similarity/homozygosity between haplotypes based on the traditional calculation of nucleotide diversity [21], which is robust to monomorphic markers. In each setting (the three allelic grouping of both the actual data and the simulated haplotype data), we generated 1000 permuted datasets, each consisting of re-sampled complete haplotypes from each allelic group, whilst maintaining allelic group sample sizes to ensure correct allele frequencies. For all comparisons, across the 1000 permuted data, the nucleotide diversity statistic was calculated. The distribution was captured as the empirical distribution function which represents the probability that the random estimates of nucleotide diversity of the permuted dataset, takes on a value less than or equal to the observed mean nucleotides diversity estimate in the actual or the simulated dataset. The proportion of the permuted dataset whose statistic values were equal to or better than the statistic of the original or simulated dataset is regarded empirical *P*-value.

The approximate likelihood coalescent-based Hotspotter software tool (‘fullopt’ module) [22] was used to estimate crossover rates (recombination rate per generation per base-pair) and detect hot spots.

In order to overcome the biases such as the ascertainment and sample selection bias that the non-random samples used here might have introduced thus making the results non-representative of the population, we displayed haplotype homozygosity patterns in only the sample-set selected from the ‘community control group’. In addition, statistical methods such as the EHH and haplotype similarities and differences were conducted on both the community controls and compared with the simulated data.

All R scripts used in this study are available from the corresponding author upon request.

## Results

### Analyses of haplotypes bearing the derived *HBB* alleles (HbC and HbS) and the ancestral HbA alleles in the KND

**Figure 2:**
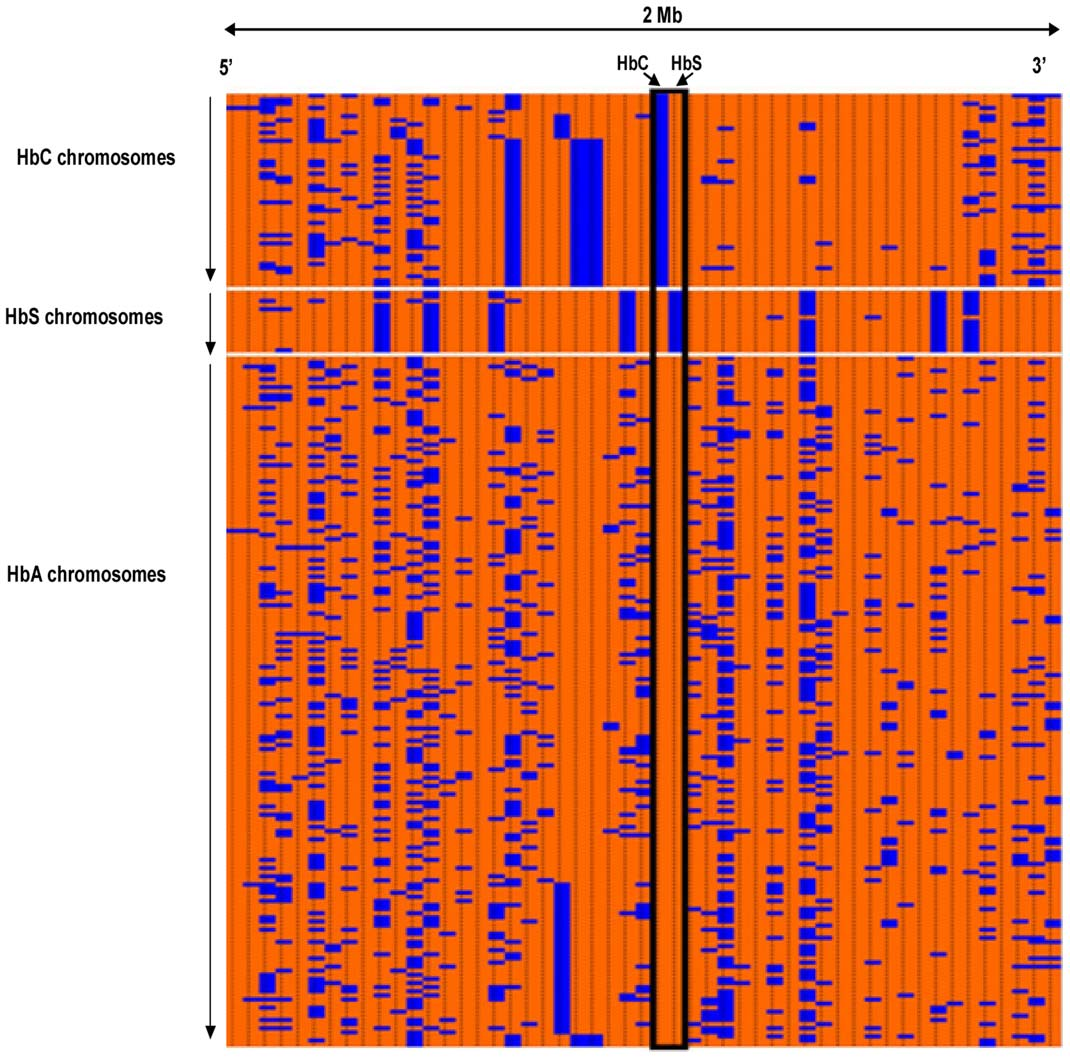
Extent of haplotype homogeneity observed in HbC, HbS and HbA chromosomes analysed. Figure 2 Illustrates of the extent of haplotype homogeneity in the haplotypes observed in HbC, HbS and HbA chromosomes in the study. The chromosomes are represented on the Y axis and 51 SNPs analysed in a 2 Mb region across the HbS locus represented on the X axis. For each of the 51 markers, major and minor alleles are coded orange and blue respectively. The 51 markers are arranged according to their position in the β globin gene region.

We analysed a 51-SNP haplotype in and around the *HBB* cluster including HbS and HbC in ‘community controls’ in the KND of Ghana. We compared derived HbS and HbC haplotypes with those bearing the ancestral HbA using the haplotype patterns formed when the data is sorted by the two derived alleles. Figure 2 illustrates the extent of homogeneity in the major haplotypes observed in derived and ancestral alleles in the KND. The HbS-bearing alleles were extensively homogeneous across the 2 Mb region with few mutations and or recombination/gene conversions occurring on a small subset of alleles. The HbC alleles remained homogeneous up to about 650 Kb and diversified with the accumulation of new mutations and or recombination mainly at the 3′end of the haplotypes. The ancestral HbA haplotypes, however, diversified essentially throughout the region.

**Figure 3:**
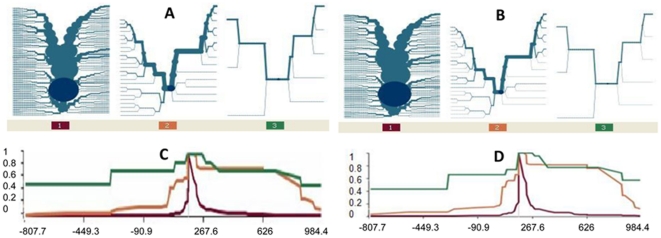
Haplotype bifurcation diagrams and extended haplotype homozygosity plots of HbC, HbS and HbA core haplotypes. Figures 3A and 3B describe the haplotype bifurcation diagrams for each core haplotype at HbC, HbS and HbA in the healthy control and simulated haplotype data respectively from the KND-Ghana. The diagrams demonstrate the extent of homozygosity in haplotypes. Diagrams one, two and three within each Figure represent HbA, HbC and HbS haplotypes respectively. Figures 3C and 3D are the extended haplotype homozygosity (EHH) plots around the core HbS/HbC loci in the healthy control and simulated haplotype data respectively. EHH scale ranges from 0 to 1: (0 implying no homozygosity, all extended haplotypes are different and 1 complete homozygosity, all extended haplotypes are the same). The green plot represents the EHH signal around the HbS locus. The orange plot represents the EHH signals around the HbC locus. And the red plot represents the lack of a pronounced EHH signal around the HbA locus.

To quantify the differences observed in homogeneity, the EHH surrounding the core haplotype (HbS and HbC loci) were calculated in both the community controls and the simulated ‘pseudo’ KND population and compared in the derived and ancestral alleles. The haplotype data are also presented as bifurcation diagrams to give a more powerful illustration of the breakdown/maintenance of haplotype structure. EHH scores for HbS haplotypes remained high both in the 5′ and 3′ ends of the region studied, with scores ranging between 0.95 and 0.5. EHH scores for HbC haplotypes were maintained between 0.95 and 0.45 from the core region up to about 650 Kb at the 5′ end of the region under study and between 0.5 and 0.4 in the 100 Kb region on the 3′ end. There was a lack of a pronounced EHH score throughout the HbA haplotypes in both the control (Figure 3C) and simulated population (Figure 3d). The scarcity of mutational branches in the HbS bifurcation diagram depicts long-range haplotype homozygosity across the region under study, although a larger sample size will be required to confirm the reliability of this finding. This finding reiterates that low-frequency alleles are generally surrounded by long haplotypes and this is a delimiting factor for the EHH test. The HbC bifurcation diagrams of the actual samples of healthy controls and the pseudo populations (Figure 3A and 3B respectively) showed a reduction in mutational branches at the 5′ ends of the bifurcation diagrams compared to the 3′ ends. This indicates systematic breakdown in the core haplotype homozygosity with distance when compared with the HbS bifurcation diagram. On the other hand, the HbA bifurcation diagram showed a high level of heterozygosity with the increased number of branches when compared to both the HbC and HbS diagrams in both the control (Figure 3A) and the simulated haplotype data (Figure 3C).

From a purely statistical view point, we explored the distribution-free property of the permutation re-sampling method to estimate nucleotide diversity as a measure of inter-allelic haplotypes differences (haplotype heterozygosity) and intra-allelic haplotype similarities (haplotype homozygosity) between the 3 haplotype groups. This idea was to compensate for the ascertainment and sampling bias resulting from the non-random sampling used in this study. First we considered the similarities/homozygosity within haplotypes in the three *HBB* variants in the healthy control haplotype set (our actual dataset). Figure S1 shows the similarities/homozygosity within haplotypes in the three *HBB* variants. On the average, for our inferred haplotype (actual haplotype) data, the probabilities that, any pair of HbC, HbS and HbA haplotypes compared were homozygous at all the loci forming the haplotypes were 0.847, 0.962 and 0.785 respectively (Figure S1). The cumulative distribution of the permuted data for the three variant groups indicate that for HbC and HbS alleles the intra-allelic mean pairwise haplotypes similarities did not obtain the values of the actual data, their values were 0.81, 0.83 , whilst the HbA haplotypes obtained a maximum estimate of 0.78.

We then considered the haplotype similarities for each *HBB* variant in the simulated data. On average, the probability estimates for haplotype similarity/homozygosity were 0.86, 0.94 and 0.79 for any pair of HbC, HbS and HbA haplotypes respectively. (Figure S2). The cumulative distribution of the permuted data for the three variant groups measured were 0.76 for HbC, 0.77 for HbS and 0.78 for HbA, all less than values obtained for the simulated data. This result implies that the observed haplotype homozygosities measured in the actual data were not chance findings, even with the sampling bias and confirms the extended haplotypes observed in HbS and HbC alleles observed in the KND

When the actual HbC haplotypes were compared with either HbS or HbA haplotypes, the mean haplotype diversity measure (haplotype differences/heterozygosity) was statistically significant (Figure S3). The mean pairwise haplotype differences/heterozygosity for HbC in comparison with HbS haplotypes was 0.26 (*P*=0.001) for actual data, and less in the permuted data (0.18). In like manner, the mean pairwise difference between actual HbC and HbA haplotypes was 0.24 (*P*=0.001), marginally greater in comparison with 0.22 for the permuted data. When comparing HbS and HbA haplotypes, there was no significant difference between the mean pairwise differences observed between the actual (*P*=0.054) and the permuted data (Figure S3). The small sample size of the HbS haplotypes is likely to have influenced this result. In the simulated data however, the average pairwise difference between HbS and HbA (0.22) was significantly higher than the difference in permuted haplotypes (0.21) (*P*=0.001) (Figure S4). Thus the haplotype heterozygosity observed between HbC and HbS/HbA alleles and between HbS and HbC/HbA haplotypes observed in the actual data set has been confirmed with the simulated data.

### Estimation of recombination rates and evidence of a ‘hotspot’ in the beta-globin gene region

**Figure 4:**
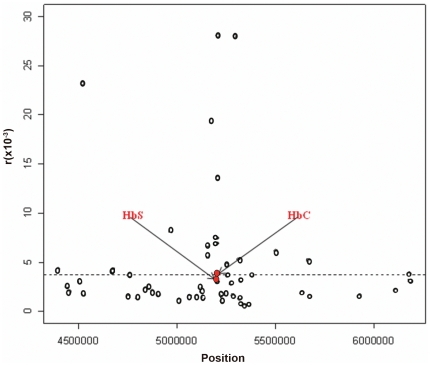
Estimates of recombination rate across the 2 Mb region of chromosome 11 analysed. Estimates of recombination rate per generation per bp (r). The dashed line indicates the average rate estimated across the region.

Using the Hotspotter software application we estimated the recombination rate per generation per base-pair for all the markers utilised in this study (Figure 4). The calculated background rate across the region is 3.73×10^−8^, which is approximately 3.4 times the genome estimate (1.1×10^−8^) (http://www.HapMap.org). Four *HBB* markers in less than 1 kb proximity from the HbS and HbC markers had recombination rates that were 5 fold greater than the background for the region, providing some evidence of a ‘recombination hot spot’. Although HbS and HbC occurred in this region, their recombination rates were below the threshold set for background recombination rate.

## Discussion

Our objectives were to describe for the first time the haplotype structure in the two β-globin variants (HbS and HbC), to throw some light on the co-evolution of the alleles in the KND of Ghana where malaria is endemic. As expected for alleles under recent selection, haplotypes carrying the derived alleles were longer than the ones bearing the ancestral allele [23], [24], [25]. HbS maintained its haplotype structure within the β-globin ‘hot spot’ and 74% of the HbC haplotypes also maintained the haplotype structure in the ‘hot spot’ region. HbC is also reported to be an allele under malaria-driven selection in parts of West Africa where it exists at high frequency [4], [5]. like other recently selected alleles such as those found in the glucose-6-phosphate dehydrogenase or lactase dehydrogenase the HbS allele would be expected to maintain its ancestral haplotypic relations over a relatively long genetic distance [24], [25], [26], [27]. The observed long range HbS haplotype is consistent with existing data that support the role of HbS as an allele that has undergone a recent selective sweep [9], [28]. The caveat to the KND HbS haplotype data, however, is that the allele frequency of HbS is low (3.8%), which is uncharacteristic of an allele under recent selection. The reason for the low HbS allele frequency is not clear but may be the result of the often lethal effect of the homozygous state. This low HbS allele frequency has been observed in malaria endemic regions where HbC [3], [4], [5] or HbE [24], [29] occur at high frequency. As per the suggestion of Modiano and colleagues, [4] and their subsequent age estimates for the two *HBB* variants [30], HbC may be older than HbS. Indeed our haplotype patterns agree with this observation. The observed long-range HbS haplotype may therefore be a reflection of the early stages of a selective sweep that may not necessarily replace the already existing allele (HbC) under selection itself. The observed allele frequency differences strengthen further the diversity in malaria driven selection [29].

Our attempts to control for potential sampling bias appear to have been successful, as similar results were obtained by testing the significance of nucleotide diversity and haplotype similarity in the haplotype data using the permutation-based metric and a simulated population based on HbS, HbC and HbA allele frequencies in population controls.

There remains some uncertainty surrounding the implications of the 5′ recombination ‘hot spot’ with regards to signals of recent selection around *HBB*. For instance, using sequence data over a 5.2 Kb region spanning HbC, Wood and colleagues in 2005 found that the recombination ‘hot spot’ was responsible for attenuation of the HbC selection signal. However, strong LD extending over 100 Kb across the β-globin recombination ‘hot spot’ has also been described in relation to positive malarial selection of the Haemoglobin E (HbE) allele in Southeast Asia [24]. In our dataset, using the observed level of homogeneity and EHH scores, we observed high haplotype homozygosity in both the HbS and the HbC haplotypes that were maintained across the ‘hot spot’ region when compared with the wild-type HbA haplotypes. Moreover, elevated recombination rates were observed about 1 Kb upstream of *HBB* and this is attributed to the existence of the ‘hot spot’ in the region. Therefore, in the haplotypes examined from the KND, the existence of the ‘hot spot’ and the high recombination rate did not reduce the effect of genetic hitchhiking on the HbS allele. HbS, therefore, maintained its signal of a strong and recent selection in the homogeneity patterns. About 74% (39/53) of the HbC alleles examined also maintained their homogeneity in this region. The HbC results compare favourably with the data obtained by Wood and colleagues in 2005 in which 61% (13/21) of the HbC haplotypes maintained the haplotype structure in the *β*-globin ‘hot spot’ region. Although about 30% of the HbC haplotypes lost structure this agrees with Wood and colleagues' conclusion of the attenuation of HbC extended haplotype. The maintenance of the HbS haplotype over a 1.5 Mb region is an indication that the ‘hot spot’ did not affect the HbS haplotype in the KND. It is likely that the strength of a selective sweep determines whether or not the ‘hot spot’ will breakdown an extended haplotype. Although it can be argued that genetic drift, bottleneck or sample sizes can also increase the frequency of a rare allele or an allele with intermediate frequency respectively, selection in HbS and HbC positive populations have been shown separately [16], [31]. In general the strength of selection in a gene region results in an extended haplotype across the region [31] as observed in the glucose-6-phosphate dehydrogenase or lactase dehydrogenase [24], [26], [25], [27]

### Conclusions

In conclusion the results of the study indicates that in the KND of Northern Ghana, the HbS and HbC alleles show clear evidence of recent positive selection. Both alleles reside on haplotypes that are significantly less diverse than those for the wild type HbA allele. They occur on different haplotypic background indicating that they might have occurred separately. They are also associated with long haplotypes: beyond 1.5 Mb for HbS and about 650 Kb for HbC. The extended HbS haplotypes observed in the populations has given more insight into how evolutionary events such as selection (recent positive selection) may be at play in Northern Ghana.

## Supporting Information

Figure S1 **Empirical distribution of mean intra-allelic haplotype similarity of the actual haplotype data.** Figure S1 is the Plot of the empirical distribution vs. mean intra-allelic haplotype similarity estimated in the permuted haplotype data of the actual data and that observed in the actual haplotype data. The empirical distribution of mean intra-allelic haplotype similarity of the actual haplotype data is shown in dotted lines and that for the permuted haplotype data is shown in smooth curve.
(DOCX)

Figure S2 **Empirical distribution of mean intra-allelic haplotype similarity of the simulated haplotype data.** Figure S2 is the Plot of the empirical distribution vs. mean intra-allelic haplotype similarity estimated in the permuted haplotype data of the simulated haplotype data and that observed in the simulated haplotype data. The distribution of mean intra-allelic haplotype similarity of the simulated haplotype data is shown in dotted lines and that for the permuted haplotype data is shown in dashed curve.
(DOCX)

Figure S3 **Empirical distribution of mean inter-allelic haplotype differences of the actual haplotype data.** Figure S3 is the Plot of the empirical distribution vs. mean inter-allelic haplotype difference estimated in the permuted haplotype data of the actual data and that observed in the actual haplotype data. The empirical distribution of mean inter-allelic haplotype differences of the actual haplotype data is shown in dotted lines and that for the permuted haplotype data is shown in smooth curve.
(DOCX)

Figure S4 **Empirical distribution of mean inter-allelic haplotype differences of the simulated haplotype data.** Figure S4 is the Plot of the empirical distribution vs. mean inter-allelic haplotype differences estimated in the permuted haplotype data of the simulated haplotype data and that observed in the simulated haplotype data. The distribution of mean intra-allelic haplotype similarity of the simulated haplotype data is shown in dotted lines and that for the permuted haplotype data is shown in the circled curve.
(DOCX)

Table S1 **Details of fifty six markers in the** ***HBB*** **region analysed.** Table S1 describes the Fifty six markers in the *HBB* region analysed with details about their rs numbers, chromosomal positions and gene reference.
(XLSX)

Table S2 **Performance of Genotyping Assays.** Table S2 describes the performance of genotyping Assays, Minor Allele Frequency (MAF) and failure rates. Assays printed in red had high failure rates and were not included in analysis.
(XLSX)

Table S3 **Genotyping data used for haplotype analysis.** Table S3 is the genotype data of the fifty one markers used in the haplotype analysis with details about their rs numbers and chromosomal positions.
(XLSX)
