# Supplementary material for: Haplotype Analyses of Haemoglobin C and Haemoglobin S and the Dynamics of the Evolutionary Response to Malaria in Kassena-Nankana District of Ghana
Source: PLoS One. 2012 Apr 10;7(4):e34565. doi: 10.1371/journal.pone.0034565 (PMC3323552; doi:10.1371/journal.pone.0034565)
Supplement: Figure S2 — Empirical distribution of mean intra-allelic haplotype similarity of the simulated haplotype data. Figure S2 is the Plot of the empirical distribution vs. mean intra-allelic haplotype similarity estimated in the permuted haplotype data of the simulated haplotype data and that observed in the simulated haplotype data. The distribution of mean intra-allelic haplotype similarity of the simulated haplotype data is shown in dotted lines and that for the permuted haplotype data is shown in dashed curve. (DOCX) [file pone.0034565.s002.docx]

Figure S2. Empirical distribution of mean intra-allelic haplotype similarity of the simulated haplotype data


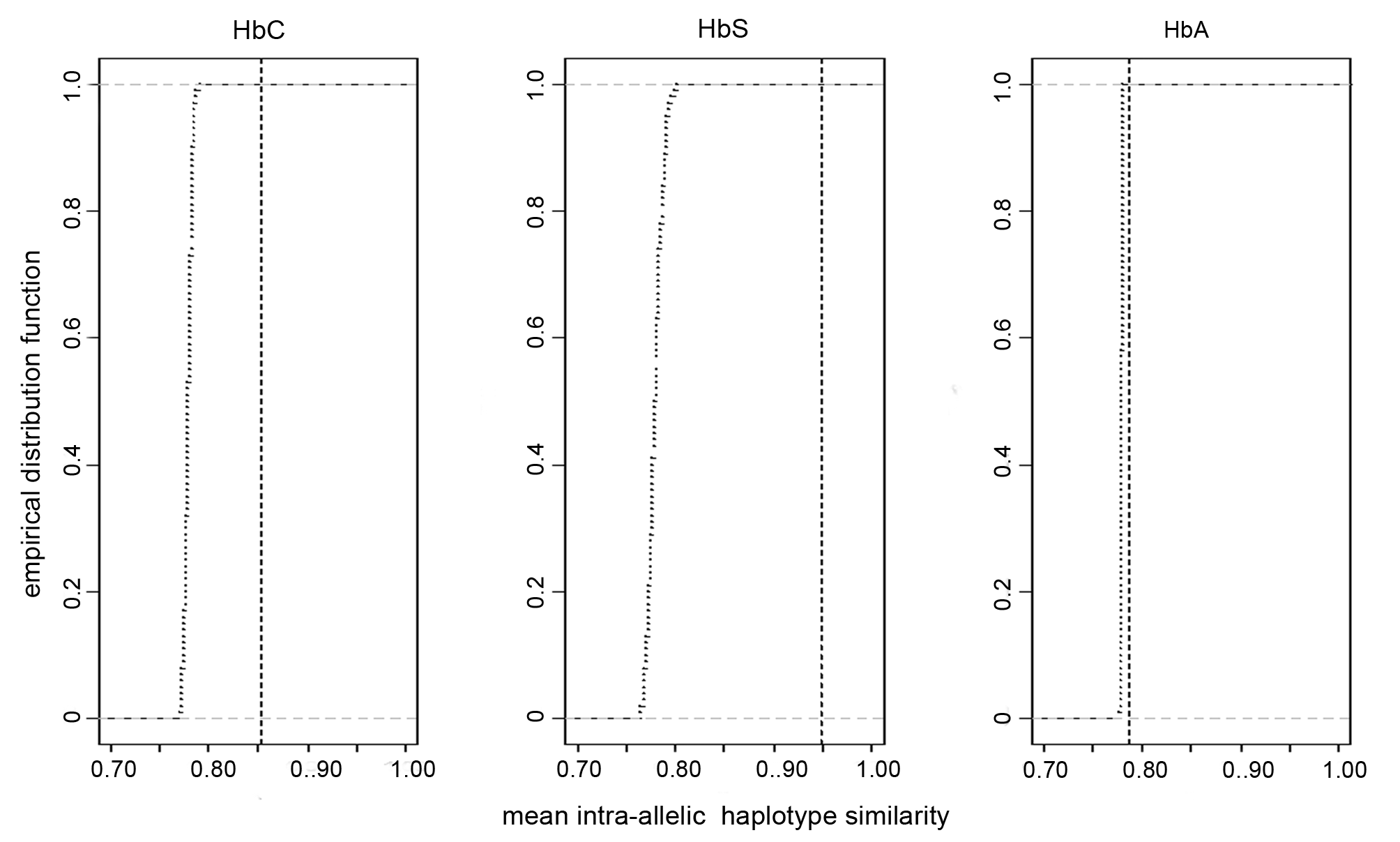


Figure S2 is the Plot of the empirical distribution vs. mean intra-allelic haplotype similarity estimated in the permuted haplotype data of the simulated haplotype data and that observed in the simulated haplotype data. The distribution of mean intra-allelic haplotype similarity of the simulated haplotype data is shown in dotted lines and that for the permuted haplotype data is shown in dashed curve.
