# Supplementary material for: Haplotype Analyses of Haemoglobin C and Haemoglobin S and the Dynamics of the Evolutionary Response to Malaria in Kassena-Nankana District of Ghana
Source: PLoS One. 2012 Apr 10;7(4):e34565. doi: 10.1371/journal.pone.0034565 (PMC3323552; doi:10.1371/journal.pone.0034565)
Supplement: Figure S3 — Empirical distribution of mean inter-allelic haplotype differences of the actual haplotype data. Figure S3 is the Plot of the empirical distribution vs. mean inter-allelic haplotype difference estimated in the permuted haplotype data of the actual data and that observed in the actual haplotype data. The empirical distribution of mean inter-allelic haplotype differences of the actual haplotype data is shown in dotted lines and that for the permuted haplotype data is shown in smooth curve. (DOCX) [file pone.0034565.s003.docx]

**Figure S3. Empirical distribution of mean inter-allelic haplotype differences of the actual haplotype data**


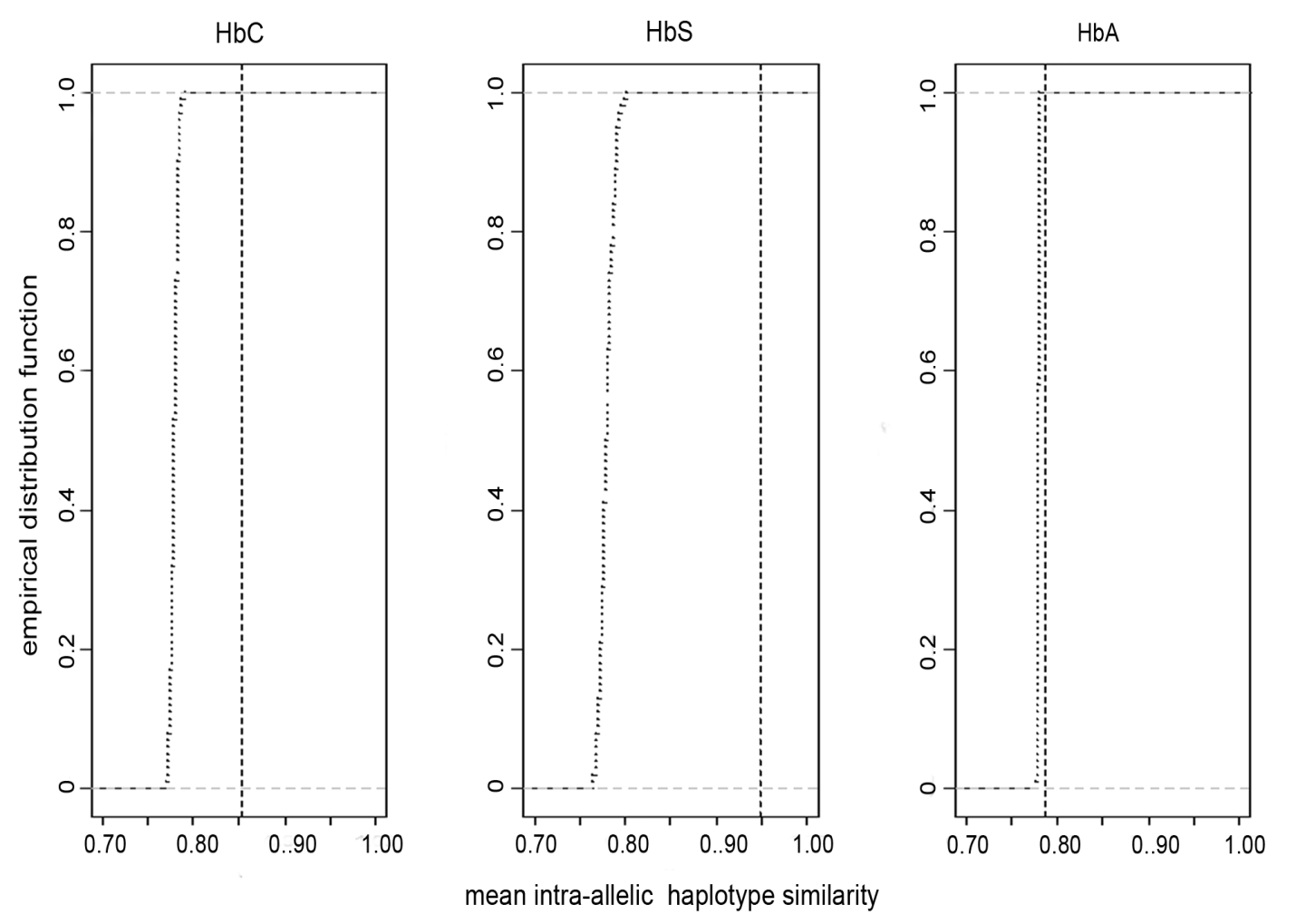


Figure S3 is the Plot of the empirical distribution vs. mean inter-allelic haplotype difference estimated in the permuted haplotype data of the actual data and that observed in the actual haplotype data. The empirical distribution of mean inter-allelic haplotype differences of the actual haplotype data is shown in dotted lines and that for the permuted haplotype data is shown in smooth curve.
