# Supplementary material for: Haplotype Analyses of Haemoglobin C and Haemoglobin S and the Dynamics of the Evolutionary Response to Malaria in Kassena-Nankana District of Ghana
Source: PLoS One. 2012 Apr 10;7(4):e34565. doi: 10.1371/journal.pone.0034565 (PMC3323552; doi:10.1371/journal.pone.0034565)
Supplement: Figure S4 — Empirical distribution of mean inter-allelic haplotype differences of the simulated haplotype data. Figure S4 is the Plot of the empirical distribution vs. mean inter-allelic haplotype differences estimated in the permuted haplotype data of the simulated haplotype data and that observed in the simulated haplotype data. The distribution of mean intra-allelic haplotype similarity of the simulated haplotype data is shown in dotted lines and that for the permuted haplotype data is shown in the circled curve. (DOCX) [file pone.0034565.s004.docx]

Figure S4. Empirical distribution of mean inter-allelic haplotype differences of the simulated haplotype data


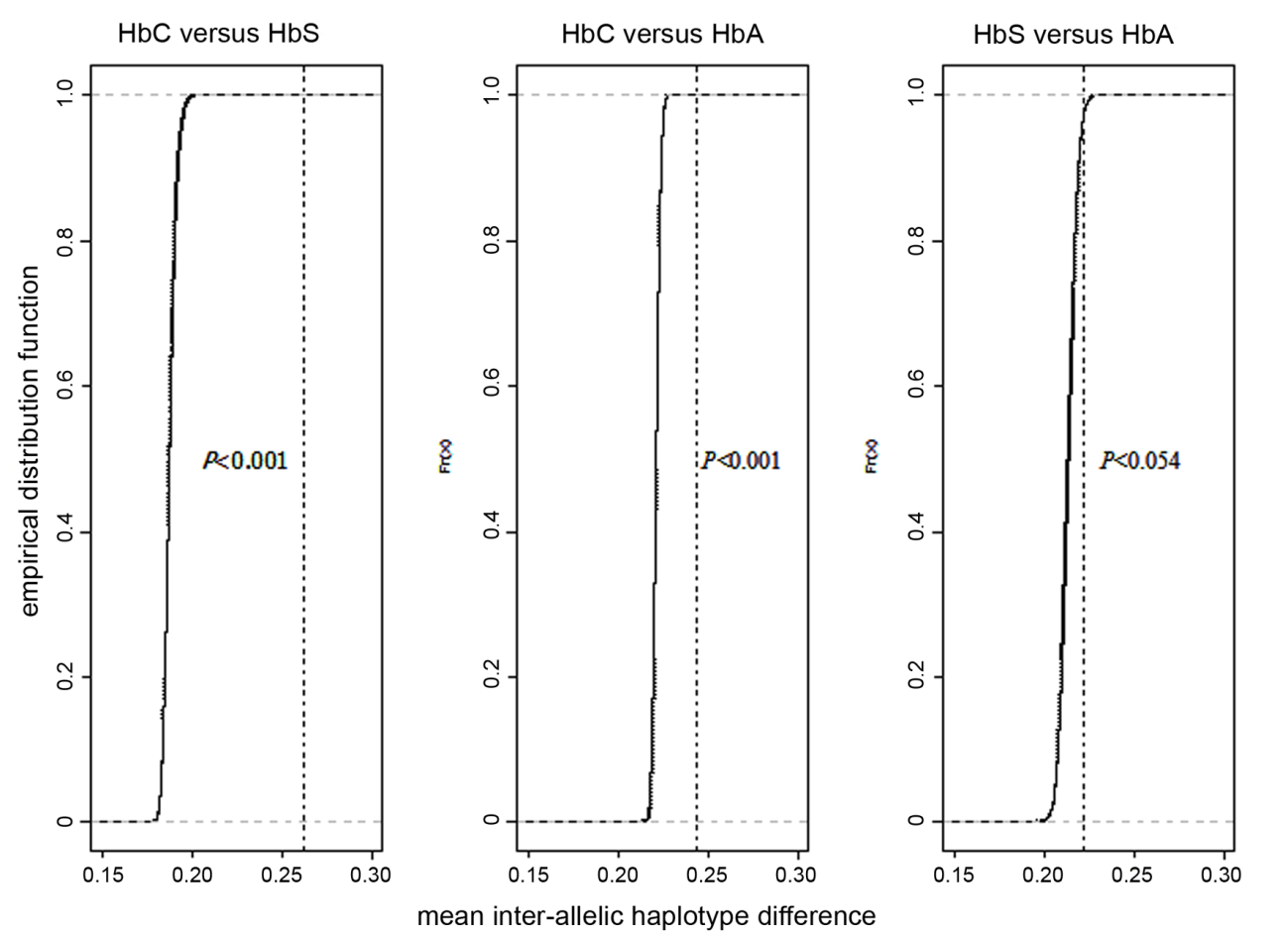


Figure S4 is the Plot of the empirical distribution vs. mean inter-allelic haplotype differences estimated in the permuted haplotype data of the simulated haplotype data and that observed in the simulated haplotype data. The distribution of mean intra-allelic haplotype similarity of the simulated haplotype data is shown in dotted lines and that for the permuted haplotype data is shown in the circled curve.
